# Big insulin-like growth factor 2-producing multiple solitary fibrous tumors treated with debulking surgery: A case report

**DOI:** 10.3389/fendo.2023.1071899

**Published:** 2023-01-20

**Authors:** Yamato Keidai, Takaaki Murakami, Nana Yamamura, Shigeru Tsunoda, Atsushi Ikeda, Koya Hida, Mototsugu Nagao, Yosuke Yamada, Ayaka Fukui, Masahito Ogura, Izumi Fukuda, Yuji Nakamoto, Kazutaka Obama, Nobuya Inagaki

**Affiliations:** ^1^ Department of Diabetes, Endocrinology and Nutrition, Graduate School of Medicine, Kyoto University, Kyoto, Japan; ^2^ Department of Surgery, Graduate School of Medicine, Kyoto University, Kyoto, Japan; ^3^ Department of Endocrinology, Metabolism and Nephrology, Graduate School of Medicine, Nippon Medical School, Tokyo, Japan; ^4^ Department of Diagnostic Pathology, Kyoto University Hospital, Kyoto, Japan; ^5^ Department of Diagnostic Imaging and Nuclear Medicine, Graduate School of Medicine, Kyoto University, Kyoto, Japan

**Keywords:** non-islet cell tumor hypoglycemia, insulin-like growth factor 2, solitary fibrous tumor, DOTATOC PET/CT, somatostatin receptor 2, octreotide, hypoglycemia unawareness, debulking surgery

## Abstract

**Background:**

Non-islet cell tumor hypoglycemia (NICTH) is a rare paraneoplastic syndrome caused by a tumor-producing high molecular weight form of insulin-like growth factor 2 (IGF2) known as big IGF2. The only curative treatment for this condition is surgical resection of the responsible tumors. However, this may not be feasible in cases with multiple metastases at diagnosis of NICTH, and no standard treatment strategy for multiple tumors has been established. The effects of pharmacological therapies including somatostatin analogs are often inefficient and remain difficult to predict.

**Case description:**

A 68-year-old man was admitted to our hospital due to impaired consciousness and severe hypoglycemia. His medical history included diagnosis of a left temporal solitary fibrous tumor (SFT) at the age of 48 years, after which local recurrent and metastatic tumors were repeatedly resected. Four years before admission, multiple intraabdominal and subcutaneous tumors were detected and, being asymptomatic, were managed conservatively. Laboratory exam on admission demonstrated hypoglycemia accompanied with low serum insulin and IGF1 levels. Computed tomography (CT) scan revealed multiple intraabdominal and subcutaneous tumors increasing in size. Serum big IGF2 was detected on immunoblot analysis, and he was diagnosed as NICTH. In addition, tumor uptake was observed on ^68^Ga-labelled 1,4,7,10-tetraazacyclododecane-N,N’,N’’,N’’’-tetraacetic acid-d-Phe^1^-Tyr^3^-octreotide positron emission tomography/CT (DOTATOC-PET/CT). Since larger tumor is more suspicious about responsible producibility of big IGF2, we planned to resect large ones preferentially and reduce the amounts of residual tumors. Debulking surgery was performed by removing eleven intraabdominal tumors; the hypoglycemia was then completely corrected. Histological analyses revealed the resected tumors to be metastases of SFT having somatostatin receptor 2 expression. In immunoblot analysis, the resected tumors were found to be positive for big IGF2; serum big IGF2 was undetectable after surgery.

**Conclusion:**

We present a case of NICTH with multiple metastatic SFTs. We strategically performed debulking surgery, which led to remission of hypoglycemia. This result demonstrates a pioneering practical solution for NICTH cases with multiple tumors. In addition, in cases of SFTs presenting with NICTH, positivity of DOTATOC-PET/CT as well as single-dose administration of octreotide may be predictive of the efficacy of somatostatin-based therapy.

## Introduction

Non-islet cell tumor hypoglycemia (NICTH) is a rare paraneoplastic syndrome caused by a tumor-producing high molecular weight form of insulin-like growth factor 2 (IGF2) known as big IGF2, which activates the insulin receptors (1). Hypoglycemia associated with hypoinsulinemia and the presence of high levels of serum big IGF2, typically in the setting of a single giant tumor, strongly supports diagnosis of NICTH (2). Although the mechanism of big IGF2 production is not fully understood, previous research suggests that overexpression of IGF2 associated with loss of imprinting is involved (3). Another report has shown that imbalance of IGF2 and proprotein convertase subtilisin/kexin type 4 (PCSK4) expression in the tumors might also be involved (4), Solitary fibrous tumor (SFT) is a rare mesenchymal neoplasm with potential to produce big IGF2 and cause hypoglycemia. Although many SFTs exhibit overproduction of IGF2 (5), only some of them acquire the ability to produce big IGF2, 4–11.5% of total cases being reported to subsequently develop symptomatic hypoglycemia (6, 7). SFTs range from benign to malignant, with 35−45% of cases showing metastasis (8).

The only curative treatment for NICTH is complete surgical excision of the responsible tumors. However, in cases with multiple tumors at diagnosis of NICTH, total resection is challenging and usually not feasible, and no standard treatment strategy for such cases has yet been established (9). In addition, although pharmacological therapies such as glucocorticoids, recombinant human GH, glucagon, and/or somatostatin analogs are also an option to alleviate hypoglycemia, the efficacy varies among cases, and long-term clinical use may be problematic due to adverse events and/or the complicated procedure (9). Moreover, the efficacies are difficult to predict (9–12).

Here we report a case of big IGF2-induced hypoglycemia in a setting of gradual growth of multiple intraabdominal and subcutaneous metastases of SFT. We strategically performed debulking surgery on the larger tumors, which resulted in clinical remission of the hypoglycemia. Furthermore, investigation in the present case suggests that ^68^Ga-labelled 1,4,7,10-tetraazacyclododecane-N,N’,N’’,N’’’-tetraacetic acid-d-Phe^1^-Tyr^3^-octreotide positron emission tomography/computed tomography (DOTATOC-PET/CT) imaging and a single-dose of octreotide administration may predict the efficacy of somatostatin analog treatment in cases of NICTH.

## Case description

A 68-year-old man was delivered to our hospital for impaired consciousness with a Glasgow Coma Scale score of E4V3M4. His serum glucose level was 28 mg/dL (1.56 mmol/L); his consciousness was completely restored by intravenous glucose administration. In his medical history, he had undergone resection of a left temporal SFT at the age of 48 years. At that time, since he had no hypoglycemic symptoms and hypoglycemia was not observed on the blood tests, no further examination about the possibility of hypoglycemia and the productivity of big IGF2 was performed in the previous hospital. At the ages of 51 and 60, he underwent repeated operations for local recurrent tumors. Again, no hypoglycemia was observed. Pathological examination showed the typical findings of SFT; the tumors were composed of spindle cells arranged in the patternless pattern. At the age of 64 years, bilateral lung metastases appeared and were treated with partial lobectomies. At that time, multiple intraabdominal and subcutaneous tumors were detected by CT scan and thereafter gradually increased in size. Based on the clinical course, we considered these tumors as metastases of SFT. However, since he was asymptomatic, he was followed up without any intervention. During the follow up, he noticed no hypoglycemic symptoms and no laboratory data showed evidence of hypoglycemia. Moreover, his dietary intake had not changed according to his and his wife’s interviews, and his weight was stable at around 50 to 52 kg (**Supplementary Table 1**).

On admission, his height was 162.0 cm; body weight, 52.0 kg; and body mass index, 19.8 kg/m^2^. On physical examination, he had palpable masses at right axilla and left hip, and he took no glucose-lowering agents. After admission to our hospital, he required continuous infusion of glucose (7–9 g/h) and a diet of 2200 kcal per day to prevent hypoglycemia. In a fasting test, after 6 hours of fasting, his plasma glucose levels dropped to 36 mg/dL (2.00 mmol/L), which was accompanied by a low serum insulin level <0.5 μU/mL, C-peptide 0.07 ng/mL, acetoacetate 16.0 μmol/L, β-hydroxybutyrate 10.7 μmol/L (**Table 1**). Notably, despite his severe hypoglycemia, he was otherwise asymptomatic. Intravenous glucagon administration (1 mg) led to an increase in plasma glucose from 36 to 87 mg/dL (2.00 to 4.83 mmol/L) in thirty minutes. The levels of counter hormones during hypoglycemia were shown in **Table 1**. A rapid ACTH test showed a peak cortisol level of 20 μg/dL 60 minutes after the infusion. His laboratory examination in the morning showed serum growth hormone level 0.09 ng/mL, IGF1 24 ng/mL (reference range, 66 to 213 ng/mL), and HbA1c 4.8%. Anti-insulin autoantibodies were negative. Renal and liver function were normal, and nutritional parameters, including albumin and cholinesterase, were not obviously decreased (**Supplementary Table 2**).

**Table 1:** Pre- and postoperative laboratory examinations.

	Early morning blood sampling			Fasting test^§^			
	preoperation	postoperation		preoperation		postoperation	
	On admission	10 days after surgery	1 month after surgery			2 weeks after surgery	
				After 6 hours	After glucagon	After 72 hours	After glucagon
Glucose [mg/dL (mmol/L)]	28 (1.56) *	90 (5.00) ^†^	103 (5.72) ^†^	36 (2.00) ^†^	87 (4.83) ^†^	55 (3.06) ^†^	57 (3.17) ^†^
Insulin [μU/mL]	<0.5	1.5	–	<0.5	<0.5	<0.5	<0.5
C-peptide [ng/mL]	<0.05	0.54	–	0.07	0.08	<0.05	<0.05
Glucagon [pg/mL]	–	–	–	26.9	–	–	–
Beta hydroxybutyrate [μmol/L]	–	10	–	10.7	–	2170	–
Acetoacetate [μmol/L]	–	6.2	–	16	–	596	–
IGF1 [ng/mL]	24^‡^	–	74^‡^	–	–	–	–
GH [ng/mL]	0.09	1.86	–	–	–	–	–
Cortisol [μg/dL]	–	–	–	11.1	–	–	–
ACTH [pg/mL]	–	–	–	31.2	–	–	–
Adrenaline [pg/mL]	–	–	–	79	–	–	–
Noradrenaline [pg/mL]	–	–	–	328	–	–	–
Dopamine [pg/mL]	–	–	–	37	–	–	–
HbA1c [%]	4.8	–	5.1	–	–	–	–

* Serum glucose level was measured. † Plasma glucose level was measured. ‡ Age and sex-matched reference range of IGF1 is 66 to 213 ng/mL. § The fasting test was performed for investigation of spontaneous hypoglycemia. After 72 hours of fasting or after confirming plasma glucose ≤45 mg/dL (2.50 mmol/L), we collected the blood samples, and glucagon (1 mg) was administered. 30 min after administration, we collected the samples again. Hyphens indicate not available. IGF1, insulin-like growth factor 1; GH, growth hormone.

**Figure 1:**
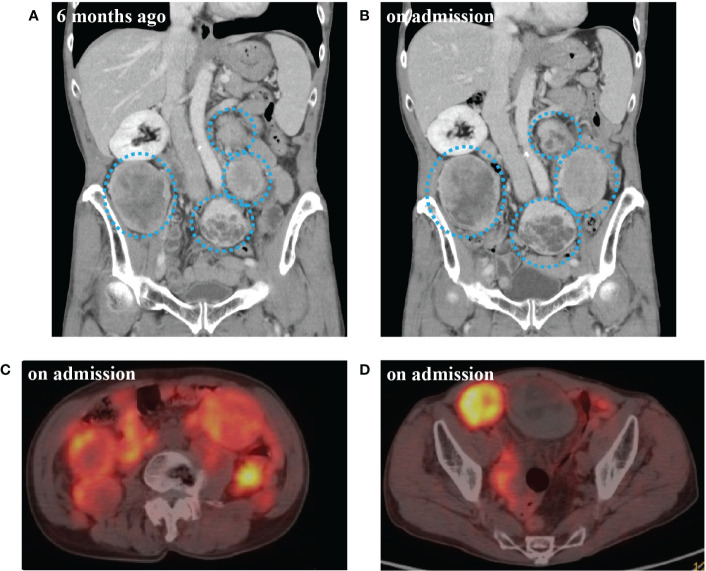
**(A)** Computed tomography (CT) scan of abdomen 6 months before admission. **(B)** CT scan at the time of admission. **(C)** ^68^Ga-labelled 1,4,7,10-tetraazacyclododecane-N,N’,N’’,N’’’-tetraacetic acid-d-Phe^1^-Tyr^3^-octreotide positron emission tomography/CT (DOTATOC-PET/CT) at abdominal level on admission. **(D)** DOTATOC-PET/CT at pelvic level on admission. CT scan of multiple intra-abdominal tumors of increasingly large size (blue dotted circle). DOTATOC PET/CT showing uptake of radiotracer in the tumors.

**Figure 2:**
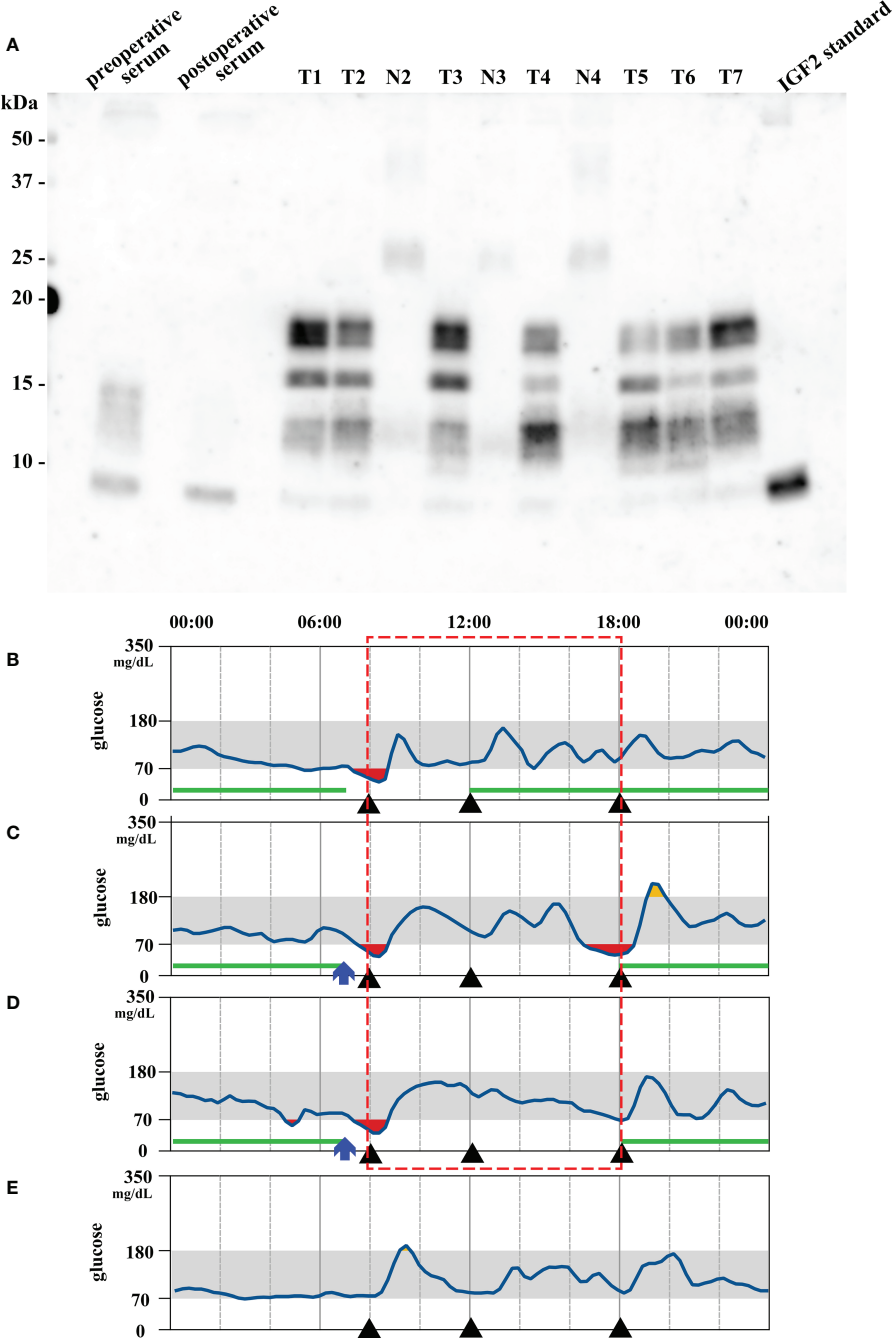
**(A)** Immunoblot analysis of insulin-like growth factor 2 (IGF2) in serum, tumors (T1–7 representing tumor #1–7) and their nearby normal tissue (N2–4 corresponding to tumor #2–4). Big IGF2 (11–18 kDa) was detected in preoperative serum and all tumor samples. The lane on the right is the IGF2 standard product. T: tumor; N: nearby normal tissue. **(B, D)** Preoperative and **(E)** 20 days postoperative continuous glucose monitoring results. **(B)** Without octreotide administration. **(C)** 50 μg Octreotide administration at 7 am (blue arrow). **(D)** 100 μg octreotide administration at 7 am (blue arrow). The red square indicates comparable period of blood glucose levels after octreotide administration. Each black triangle represents meal intake. The green lines indicate the period during which 9 g/h of glucose was intravenously administered.

Due to past history of SFT, we performed a CT scan on admission. CT scan revealed 14 intraabdominal tumors with maximum size 9.1 cm and 4 small subcutaneous tumors maximum size 3.3 cm at right axilla and left hip. The tumors were increased in size compared to six months before (**Figures 1A, B**). These findings in combination with the laboratory results suggested that metastatic lesions of SFT were producing big IGF2 to cause hypoglycemia. Immunoblot analysis revealed serum big IGF2, which established a clinical diagnosis of NICTH (**Figure 2A**). As larger tumors are more likely to produce big IGF2 and be more responsible for the hypoglycemia, we planned to perform debulking surgery on the larger tumors preferentially, seeking to reduce the level of big IFG2 secretion as well as the total amount of residual tumors, and significantly reduce hypoglycemia.

Concurrently, we also investigated a pharmacological treatment option. DOTATOC-PET/CT showed high uptakes of the radiotracer in all of the intraabdominal and subcutaneous lesions, indicating the expression of somatostatin receptors (SSTRs), mainly SSTR 2 and 5 (**Figures 1C, D**). Accordingly, the efficacy of octreotide to improve hypoglycemia was examined in the preoperative period. Single-dose subcutaneous octreotide administration was found by continuous glucose monitoring (CGM) system to increase the postprandial blood glucose level (**Figures 2B–D**).

**Figure 3:**
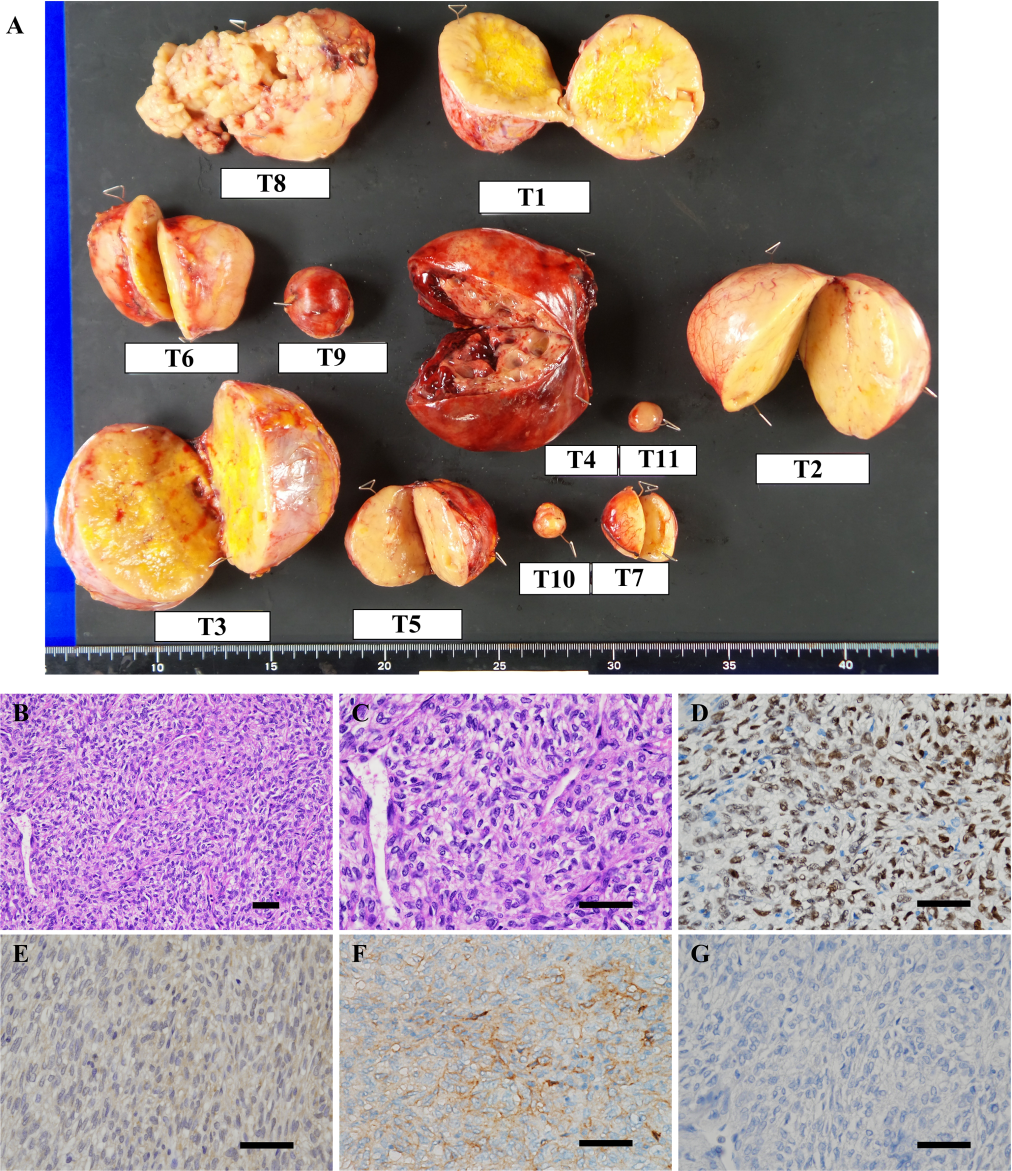
**(A)** Macroscopic view of resected tumors. T1–11 represent tumor #1–11. **(B–G)** Microscopic findings of the representative resected tumor. **(B, C)** Hematoxylin and eosin staining showed that the tumor was composed of spindle to ovoid cells forming the patternless pattern. Immunohistochemical stains of tumor #10 show tumor cells to be positive for **(D)** signal transducer and activator of transcription 6 (STAT6), **(E)** IGF2, **(F)** somatostatin receptor 2 (SSTR2) and negative for **(G)** SSTR5. Scale bar: 50 μm.

In the surgery, five of the largest intraabdominal tumors (tumor #1–4, #8 in **Figure 3A**) and six small tumors accessible from the abdominal operative field (tumor #5–7, #9–11 in **Figure 3A**) were resected. Histopathological examination revealed spindle to ovoid shaped signal transducer and activator of transcription 6 (STAT6)-positive tumor cells, compatible with the metastatic SFTs (**Figures 3B–D**). Additional immunohistochemical studies showed positive immunostaining for IGF2 and SSTR2 and negative immunostaining for SSTR5 in the tumor cells (**Figures 3E–G**). The MIB-1 index was 5%. In order to seek which tumor was responsible for the production of big IGF2 in the resected ones, we performed immunoblot analyses. The immunoblot analyses of 7 of the 11 resected tumors, for which samples were available, revealed big IGF2 production in all of them (**Figure 2A**). After the surgery, hypoglycemia was resolved even after discontinuation of glucose infusion (**Figure 2E**). Consistently, serum big IGF2 was null one week after the surgery (**Figure 2A**). In addition, two weeks after the surgery, 72-hour fasting showed that plasma glucose levels no longer dropped to 55 mg/dL (3.06 mmol/L) with high ketone body levels; acetoacetate was 596 μmol/L and β-hydroxybutyrate was 2,170 μmol/L (**Table 1**). Moreover, no apparent hypoglycemia was found by CGM. One month after the surgery, the HbA1c level was 5.1%, and the serum IGF1 concentration was restored to the levels of normal range (**Table 1**). Subsequently, hypoglycemia was not observed for three-month follow-up and the HbA1c level increased to 5.7%. These findings indicated that the subcutaneous tumors were considered to have a small or negligible contribution to hypoglycemia.

## Discussion

We present a patient having NICTH associated with multiple metastatic SFTs, in which debulking surgery successfully reduced serum big IGF2 and restored normoglycemia. In addition, the tumors were found to be positive for DOTATOC-PET/CT, and preoperative octreotide administration was able to increase blood glucose levels, which may indicate predictability of the efficacy of somatostatin analogs in the treatment of NICTH.

In the present case, we performed debulking surgery with the strategy of resecting larger tumors and reducing the tumor volume, which resulted in reduced serum big IGF2 and remission of hypoglycemia. This strategy may be useful for the management of hypoglycemia in NICTH cases with multiple tumors. Although the only curative treatment of NICTH is complete surgical resection of the responsible tumors (9), it is challenging and not feasible in most of cases in which the tumors show multiple metastasis at NICTH diagnosis. Hence, optimal treatment strategy for multiple metastatic tumors with NICTH remains uncertain (9). Moreover, in cases of NICTH with multiple SFTs, it is critical to determine which of the tumors is most responsible for the production of big IGF2 and the consequent development of hypoglycemia. Since most big IGF2-producing tumors that develop in NICTH are reported to be large in size (2), it is likely that the larger ones produce big IGF2 rather than the smaller ones, and should therefore be high priority targets for resection. Even in cases of multiple tumors producing big IGF2, it seems beneficial to reduce tumor volume as far as possible, especially since an association between tumor volume and development of hypoglycemia has been reported (9). Although there are previous reports demonstrating the effectiveness in cases of NICTH of debulking treatment, including subtotal tumor resection or embolization (13–15), preferred strategy for an optimal resection in multiple tumors has not been discussed. As a result of the strategic surgery in the present case, serum big IGF2 became null and the hypoglycemia was resolved. Therefore, our case may well demonstrate a pioneering actual solution for NICTH cases with multiple tumors.

As pharmacological approaches to alleviate hypoglycemia due to NICTH, glucocorticoids, recombinant human GH, and glucagon preparations have been reported (9). But their clinical use is impeded by the poor efficacy, adverse events, and/or clinical availability (9). Somatostatin analogs also have been suggested, but their efficacy varies across cases, and clinical predictors of the therapeutic response have not been established (10–12). In present case, we found that the tumors showed uptakes on DOTATOC-PET/CT, and histopathological analyses confirmed the expression of SSTR2 on the resected tumor cells. We therefore investigated for efficacy of somatostatin analogs as a therapeutic option for residual hypoglycemia that might occur after debulking surgery. Indeed, single-dose administration of octreotide was shown to improve blood glucose levels by CGM. Taken together with the previous reports showing that some SFTs show uptake on SSTR-targeted imagings such as ^111^In-labelled octreotide scintigraphy and DOTATOC-PET/CT (12, 16, 17), these findings in our case strongly suggest the usefulness of somatostatin analogs as a treatment option for the NICTH cases with the tumor positivity of SSTR-targeted imagings. Contrariwise, a previous case report found that tumor positivity on SSTR-targeted imaging alone did not accurately predict successful glycemic control by somatostatin analog, although only one case showed such inconsistency (12). Thus, further investigation is required to establish predictive utilities of SSTR-targeted imagings for the responsiveness to somatostatin analogs in cases of NICTH. At the same time, it is also considered that the addition of single-dose octreotide administration is a reasonable way to further confirm the glycemic responsiveness of somatostatin analogs. In cases with SFT presenting with NICTH, the combination of positivity of DOTATOC-PET/CT and glycemic response to a single-dose administration of octreotide may well be informative in exploring the efficacy of somatostatin-based therapy.

In the present case, the patient was asymptomatic before the development of severe hypoglycemia, suggesting hypoglycemia unawareness. Unawareness of the neurogenic symptoms can be induced by repeated and prolonged hypoglycemic episodes (18), and can result in delayed diagnosis and severe hypoglycemia (19, 20). As big IGF2 was detected in all metastatic lesions in our case, it is plausible that it was produced from the first appearance of the intraabdominal and subcutaneous metastases. Thus, the gradual increase of big IGF2 over a long period of at least four years might well have contributed to hypoglycemia unawareness. In SFT cases, if not all tumors can be completely resected, asymptomatic patients are usually followed up without any intervention (21). However, during this follow-up period, the hypoglycemia unawareness would mask the onset of NICTH and delay the prompt intervention in cases with big IGF2-producing SFTs. Furthermore, the timing of blood tests may also affect the detection of hypoglycemia. In the present case, in blood tests before admission, we checked only casual blood glucose levels, not fasting ones. This might mask the existence of hypoglycemia and make it challenging to detect hypoglycemia in the outpatient setting. Therefore, early vigilance of hypoglycemia is required in cases of SFTs potentially producing big IGF2, especially at the stage of increasing tumor volume. To recognize the appearance of hypoglycemia earlier, trends of HbA1c and IGF1 levels as well as fasting blood glucose could be useful. At the same time, exploring the big IGF2 production may also be useful using the serum or biopsy to establish the diagnosis of big IGF2-producing SFTs.

This case report has several limitations. First, it is unknown when the big IGF2 secretion of tumors and hypoglycemia initially appeared in the present case. This may emphasize the clinical difficulty and importance of early vigilance of NICTH. Second, the relationship between DOTATOC uptakes and octreotide efficacy should be further investigated in larger scale clinical studies of SFT cases presenting with NICTH. Finally, the long-term prognosis after debulking surgery is still unknown. Extended follow-up studies should be performed in multiple metastatic SFT cases.

In conclusion, we present a case of NICTH with multiple metastatic SFTs. We strategically performed debulking surgery, which led to reduced serum big IGF2 and clinical remission of hypoglycemia. In addition, we demonstrated positivity of DOTATOC-PET/CT and a favorable glycemic response to single-dose administration of octreotide. In NICTH cases with multiple tumors, the treatment strategy of resecting larger tumors and reducing the tumor volume can be an option to be considered. Moreover, in cases with SFT presenting with NICTH, a positivity of DOTATOC-PET/CT may well be informative for the efficacy of somatostatin-based therapy.

## Data availability statement

The original contributions presented in the study are included in the article/**Supplementary Material**. Further inquiries can be directed to the corresponding authors.

## Ethics statement

The studies involving human participants were reviewed and approved by the institutional review boards of Kyoto University. The patients/participants provided their written informed consent to participate in this study. Written informed consent was obtained from the individual(s) for the publication of any potentially identifiable images or data included in this article.

## Author contributions

YK and TM contributed to the conception and design of the study and wrote the manuscript. NY, ST, AI, KH, MO and KO took responsibility for patient management. YY and AF performed the histological diagnosis of the tumors. MN and IF performed histological and biochemical analyses. YN performed imaging analyses. NI contributed to overall relevant discussions and reviewed the manuscript. All authors contributed to the article and approved the submitted version.
