# Supplementary material for: Big insulin-like growth factor 2-producing multiple solitary fibrous tumors treated with debulking surgery: A case report
Source: Front Endocrinol (Lausanne). 2023 Jan 20;14:1071899. doi: 10.3389/fendo.2023.1071899 (PMC9895772; doi:10.3389/fendo.2023.1071899)
Supplement: Supplementary file 1 [file Table_1.docx]

Supplementary Material

Big Insulin-like Growth Factor 2-Producing Multiple Solitary Fibrous Tumors Treated with Debulking Surgery: A Case Report

Yamato Keidai, Takaaki Murakami^*^, Nana Yamamura, Shigeru Tsunoda, Atsushi Ikeda, Koya Hida, Mototsugu Nagao, Yosuke Yamada, Ayaka Fukui, Masahito Ogura, Izumi Fukuda, Yuji Nakamoto, Kazutaka Obama, Nobuya Inagaki^*^

*** Correspondence:** Takaaki Murakami E-mail: tmurakam@kuhp.kyoto-u.ac.jp or Nobuya Inagaki E-mail: inagaki@kuhp.kyoto-u.ac.jp

**Supplemental table 1. Medical history of the patient**

| Age (years) | Tumor findings | Treatments | Exploration of big IGF2 | Presentation of hypoglycemia | |
| --- | --- | --- | --- | --- | --- |
|  |  |  |  | symptoms | blood test |
| 48 | Left temporal SFT (primary lesion) | Resection | Not analyzed | Asymptomatic | No hypoglycemia |
| 51 | Left temporal SFT (local recurrence) | Resection | Not analyzed | Asymptomatic | No hypoglycemia |
| 60 | Left temporal SFT (local recurrence) | Resection | Not analyzed | Asymptomatic | No hypoglycemia |
| 64 | Bilateral lung metastases of SFT | Resection | Not analyzed | Asymptomatic | No hypoglycemia |
|  | Multiple intraabdominal and subcutaneous tumors were first detected by CT scan. | No intervention | Not analyzed | Asymptomatic | No hypoglycemia |
| 68 | Multiple intraabdominal and subcutaneous tumors increased in size. | Debulking surgery | Big IGF2 was detected in blood and tumor samples | Impaired consciousness | Severe hypoglycemia |

**Supplemental table 2.** **Laboratory data on admission**

| AST | 25 | IU/L |
| --- | --- | --- |
| ALT | 17 | IU/L |
| γ-GTP | 20 | IU/L |
| Cr | 0.53 | mg/dL |
| eGFR | 116 | mL/min/1.73m^2^ |
| TP | 5.9 | g/dL |
| Alb | 3.4 | g/dL |
| ChE | 194 | U/L |
